# Correction: Corrosion and tribocorrosion performance of multilayer diamond-like carbon film in NaCl solution

**DOI:** 10.1039/d1ra90176a

**Published:** 2021-12-23

**Authors:** Mingjun Cui, Jibin Pu, Jun Liang, Liping Wang, Guangan Zhang, Qunji Xue

**Affiliations:** State Key Laboratory of Solid Lubrication, Lanzhou Institute of Chemical Physics, Chinese Academy of Sciences Lanzhou 730000 China lpwang@licp.cas.cn gazhang@licp.cas.cn +86 931 4968163 +86 931 4968080; Key Laboratory of Marine Materials and Related Technologies, Ningbo Institute of Materials Technology and Engineering, Chinese Academy of Sciences Ningbo 315201 China; University of Chinese Academy of Sciences Beijing 100039 China

## Abstract

Correction for ‘Corrosion and tribocorrosion performance of multilayer diamond-like carbon film in NaCl solution’ by Mingjun Cui *et al.*, *RSC Adv.*, 2015, **5**, 104829–104840, DOI: 10.1039/C5RA21207C.

The authors regret that the scale bars for the SEM image in Fig. 3a and c were mislabelled. The correct scale bar should be 100 μm, and the corresponding SEM images are shown below.
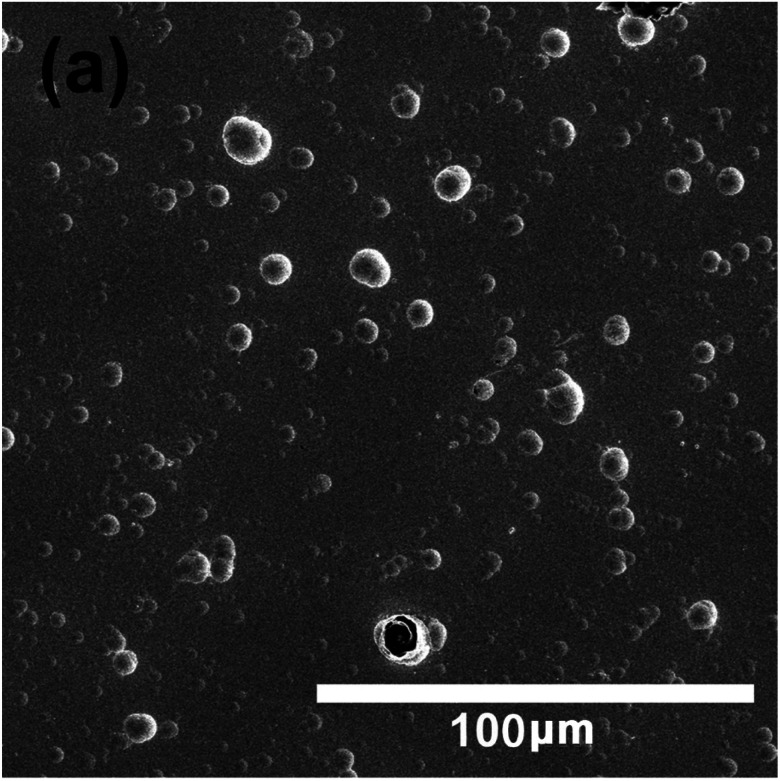


Fig. 3a SEM surface morphology for single layer DLC film.
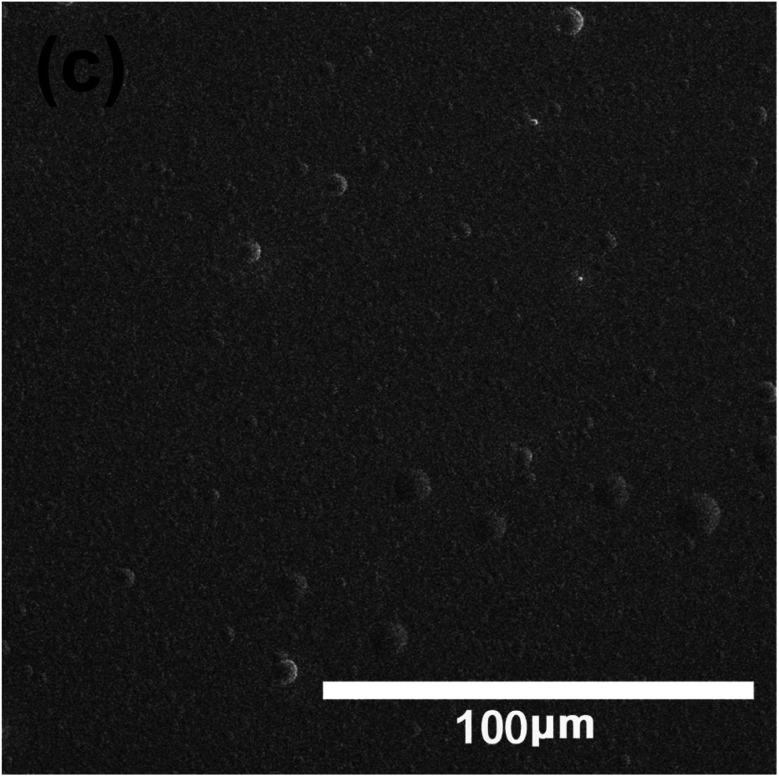


Fig. 3c SEM surface morphology for multilayer DLC film.

The Royal Society of Chemistry apologises for these errors and any consequent inconvenience to authors and readers.

## Supplementary Material

